# Characteristics and Survival Among Patients With Cardiogenic Shock Undergoing Mitral Transcatheter Edge-to-Edge Repair

**DOI:** 10.1016/j.jacadv.2025.102355

**Published:** 2025-11-18

**Authors:** Leila Haghighat, Richard Cheng, Christopher F. Barnett, Samuel B. Brusca, Jeong-Gun Park, Anthony P. Carnicelli, Andrew H. Nguyen, Raunak M. Nair, David A. Morrow, Connor G. O’Brien

**Affiliations:** a Division of Cardiology, Department of Medicine, University of California San Francisco, San Francisco, California, USA; b Sutter Health, Palo Alto Medical Foundation, Palo Alto, California, USA; c Division of Cardiology, Department of Medicine, University of California Los Angeles, Los Angeles, California, USA; d Levine Cardiac Intensive Care Unit, TIMI Study Group, Cardiovascular Division, Department of Medicine, Brigham and Women’s Hospital and Harvard Medical School, Boston, Massachusetts, USA; e Divison of Cardiology, Department of Medicine, Medical University of South Carolina, Charleston, South Carolina, USA; f Department of Internal Medicine, Inova Fairfax Hospital, Falls Church, Virginia, USA; g Cleveland Clinic, Vascular and Thoracic Institute, Cleveland, Ohio, USA

**Keywords:** cardiogenic shock, mitral regurgitation, transcatheter therapies

**What is the clinical question being addressed?**

In patients with cardiogenic shock where mitral regurgitation is the primary cause or a major contributing factor, what is the survival rate following treatment with M-TEER, and which clinical characteristics are associated with mortality?

**What is the main finding?**

Seventy-five percent of patients with treated with M-TEER survived to hospital discharge, including those requiring advanced ICU support. In-hospital mortality was associated with higher SCAI shock stage, elevated lactate levels, and concomitant distributive shock.

Mitral transcatheter edge-to-edge repair (M-TEER) improves survival among outpatients with medically optimized, hemodynamically significant mitral regurgitation (MR).^1^ However, data on M-TEER among patients with cardiogenic shock (CS) remain scant. Current understanding from a retrospective registry study identified the association between procedural success of M-TEER in CS and lower 1-year all-cause mortality.^2^ A meta-analysis predominantly from this study reported low mortality rates among CS patients receiving M-TEER, including 11% during hospitalization, and 36% at 1 year.^3^ Detailed shock characteristics were not available.

## Methods

To better understand the utility of M-TEER in CS and identify characteristics associated with survival, we leveraged the Critical Care Cardiology Trials Network (CCCTN) registry. CCCTN is an investigator-initiated research network of predominantly North American cardiac intensive care units (CICUs) coordinated by TIMI Study Group (Boston, MA). The CCCTN protocol was approved by the Institutional Review Board at Mass General Brigham and each participating center. Registry methods have been reported.^4^ All CS patients who underwent M-TEER were evaluated. The primary outcome was in-hospital survival. We compared survivors and nonsurvivors for baseline demographics, shock characteristics, and ICU therapies. Categorical variables are reported as percentages with frequency to identify rare missingness and were compared using the Fisher exact test. Continuous variables are reported as median (IQR) and were compared using Wilcoxon rank sum tests. Patients receiving renal replacement therapy were excluded from reporting of estimated glomerular filtration rate. This study was completed in accordance with Strengthening the Reporting of Observational Studies in Epidemiology (STROBE) reporting guidelines.

## Results

**Figure 1:**
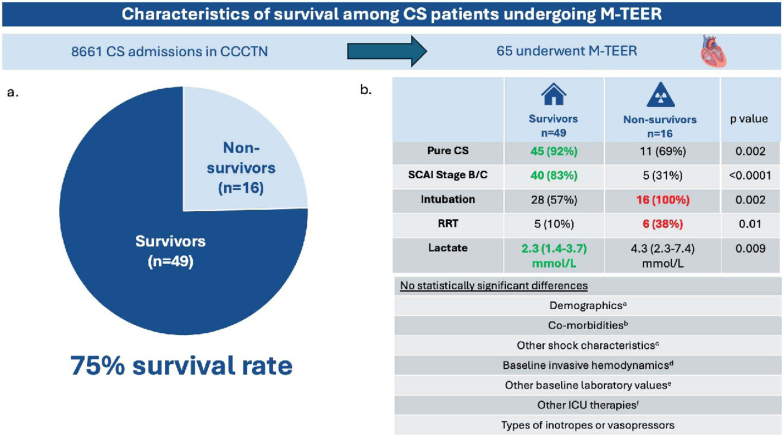
**Characteristics of Survival Among CS Patients Undergoing M-TEER** (A) Rate of survival to hospital discharge among patients with isolated cardiogenic shock or mixed vasodilatory cardiogenic shock undergoing mitral transcatheter edge-to-edge repair. (B) Characteristics of survivors vs nonsurvivors with cardiogenic shock who underwent transcatheter edge-to-edge repair. Categorical variables are reported as number (%). Continuous variables are reported as median (IQR). ^a^Demographics in analysis include age, weight, body mass index, sex, and race. ^b^Comorbidities in analysis include smoking history, hypertension, coronary artery disease, cerebrovascular disease, peripheral artery disease, atrial fibrillation, severe valvular disease, pulmonary hypertension, heart failure, diabetes, chronic kidney disease, pulmonary disease, liver disease, dementia, and cancer. ^c^Other shock characteristics in analysis include left ventricular ejection fraction at shock presentation, maximum number of inotropes or vasopressors, and Sequential Organ Failure Assessment (SOFA) score. ^d^Baseline hemodynamics in analysis include heart rate, systolic blood pressure, mean arterial pressure, right atrial pressure, pulmonary artery systolic and diastolic pressures, pulmonary capillary wedge pressure, and cardiac index. ^e^Baseline laboratory values in analysis include hemoglobin, creatinine, estimated glomerular filtration rate, and arterial pH. ^f^Other ICU therapies in analysis include noninvasive positive pressure ventilation, high-flow oxygen, inhaled pulmonary vasodilator, invasive hemodynamic monitoring, target temperature management, and mechanical circulatory support. CCCTN = Critical Care Cardiology Trials Network; CS = cardiogenic shock; ICU = intensive care unit; RRT = renal replacement therapy; M-TEER = mitral transcatheter edge-to-edge repair; SCAI = Society for Cardiovascular Angiography and Interventions.

CICU admissions (n = 34,686) between 2017 and 2024 were analyzed. Of 8,661 CS admissions, 115 (1.3%) had MR as a primary or major contributor to CS and 65 patients underwent M-TEER; only 9 of which were acute myocardial infarction cardiogenic shock (AMI-CS) (Figure 1). Most patients were Caucasian (80%) and male (60%) with history of coronary artery disease (48%), atrial fibrillation (62%), severe valvular disease (69%), and heart failure (69%). 3.1% were Society for Cardiovascular Angiography and Interventions (SCAI) shock class B, 67.2% C, 26.6% D, and 3.1% E. Fifty percent had lactate of ≥3 mmol/L; 65% received ≥2 concurrent inotropes or vasopressors, and 34% received one. The majority of CS M-TEER patients survived to hospital discharge (75%). Of the 16 nonsurvivors, 6 died from CS, 5 from mixed shock, 2 from acute respiratory failure, 1 from septic shock, 1 from ventricular free wall rupture, and 1 from pulseless electrical activity arrest.

Despite no statistically significant differences in baseline demographics between survivors and nonsurvivors, survivors trended toward being younger (73 [65-78] vs 75 [68-83] years, *P* = 0.27), with less heart failure with preserved ejection fraction [HFpEF] (32% [12/37] vs 63% [5/8], *P* = 0.36). Pure CS rather than mixed shock was more common among survivors (92% [45/49] vs 69% [11/16], *P* = 0.03; Figure 1). Admission SCAI shock stage was lower among survivors (SCAI B/C 83% [40/48] vs 31% [5/16], SCAI D/E 17% [8/48] vs 69% [11/16], *P* = 0.0002), with no significant difference in Sequential Organ Failure Assessment score (7 [5-10] vs 8 [7-10], *P* = 0.20). Baseline laboratories were similar between survivors and nonsurvivors, including hemoglobin (11.7 [10.1-13.0] vs 11.9 [10.0-13.5] mg/dL), estimated glomerular filtration rate (36 [22.2-58.2] vs 36 [35.1-46.2] mL/min/1.73 m^2^), and arterial pH (7.4 [7.3-7.4] vs 7.4 [7.2-7.4]); although admission lactate was lower in survivors (2.3 [1.4-3.7] vs 4.3 [2.3-7.4] mmol/L, *P* = 0.009). Survivors were less likely to be mechanically ventilated (57% [28/49] vs 100% [16/16], *P* = 0.001) or require renal replacement therapy (10% [5/49] vs 38% [6/16], *P* = 0.02). Rates of mechanical circulatory support were 33% (16/49) vs 56% (9/16) (*P* = 0.14). Survival was 76% (16/21) among those supported with intra-aortic balloon pump (IABP), 25% (1/4) with venoarterial extracorporeal membrane oxygenation (VA-ECMO), and 0% (0/2) with Impella.

## Discussion

Uniquely, data from CCCTN allowed us to evaluate previously unreported detailed shock characteristics and therapies among those with CS undergoing M-TEER. The major findings of this cohort study are 3-fold: 1) despite being severely ill, most patients (75%) survived to hospital discharge; 2) CS survivors who underwent M-TEER frequently required advanced ICU therapies; 3) greater SCAI shock stage and higher lactate appear associated with in-hospital mortality. Consistent with contemporary epidemiology of CS, our findings show that the development of concomitant distributive shock carries greater mortality.

Limitations of our study include small sample size, which is reflective of the off-label use of M-TEER. Additionally, patients who underwent M-TEER may have been selected based on a global assessment of prognosis. The nonrandomized design precludes comparative assessment of the benefit of M-TEER.

Our findings suggest promise for M-TEER in carefully selected patients with CS, 23% of whom have valvular heart disease contribute to their shock.^5^ Despite high mortality rates associated with CS and significant MR, in-hospital survival is favorable among CS patients selected for M-TEER. Given the high rates at which survivors utilized advanced ICU therapies, needing these therapies should not deter consideration of M-TEER. Discerning patients with pure CS before developing superimposed vasoplegia may better identify candidates for M-TEER; this finding warrants further investigation. Further refinement of prognosticators may guide quality metrics for M-TEER and eventually define a category of patients in whom M-TEER can be salvage therapy.

## Funding support and author disclosures

Dr Barnett performs research for Merck and Pfizer and receives consulting fees from 10.13039/100000046Abbott, Johnson & Johnson MedTech, and 10.13039/100015345Zoll. Dr Brusca consults and performs research for 10.13039/100004331Johnson & Johnson. Dr Cheng receives consulting fees from 10.13039/100000046Abbott and 10.13039/100006520Edwards. Dr Morrow receives research grant support to TIMI Study Group through Brigham and Women’s Hospital from 10.13039/100001316Abbott Laboratories, 10.13039/100020297Abiomed, 10.13039/100002429Amgen, 10.13039/100020132Anthos Therapeutics, 10.13039/100004325AstraZeneca, 10.13039/501100022274Daiichi Sankyo, 4TEEN4, 10.13039/100014130Intarcia, 10.13039/100005565Janssen, 10.13039/100004334Merck, 10.13039/100004336Novartis, 10.13039/100004319Pfizer, Poxel, 10.13039/100009857Regeneron, 10.13039/100004337Roche, and 10.13039/100004340Siemens; and also receives consulting fees from 10.13039/100001316Abbott Laboratories, 10.13039/100004334Merck, 10.13039/100004336Novartis, 10.13039/100009857Regeneron, and 10.13039/100016545Roche Diagnostics. Dr O’Brien receives consulting fees from Johnson & Johnson MedTech. All other authors have reported that they have no relationships relevant to the contents of this paper to disclose.
